# Effects of Computer-Aided Manufacturing Technology on Precision of Clinical Metal-Free Restorations

**DOI:** 10.1155/2015/619027

**Published:** 2015-10-18

**Authors:** Ki-Hong Lee, In-Sung Yeo, Benjamin M. Wu, Jae-Ho Yang, Jung-Suk Han, Sung-Hun Kim, Yang-Jin Yi, Taek-Ka Kwon

**Affiliations:** ^1^Department of Prosthodontics, School of Dentistry and Dental Research Institute, Seoul National University, Daehak-ro, Jongno-gu, Seoul 110-749, Republic of Korea; ^2^Division of Advanced Prosthodontics, UCLA School of Dentistry, Los Angeles, CA 90095, USA; ^3^Department of Dentistry, St. Vincent Hospital, Catholic University of Korea, Ji-dong, Paldal-gu, Suwon 442-723, Republic of Korea

## Abstract

*Purpose*. The purpose of this study was to investigate the marginal fit of metal-free crowns made by three different computer-aided design/computer-aided manufacturing (CAD/CAM) systems. *Materials and Methods*. The maxillary left first premolar of a dentiform was prepared for all-ceramic crown restoration. Thirty all-ceramic premolar crowns were made, ten each manufactured by the Lava system, Cercon, and Cerec. Ten metal ceramic gold (MCG) crowns served as control. The marginal gap of each sample was measured under a stereoscopic microscope at 75x magnification after cementation. One-way ANOVA and the Duncan's post hoc test were used for data analysis at the significance level of 0.05. *Results*. The mean (standard deviation) marginal gaps were 70.5 (34.4) *μ*m for the MCG crowns, 87.2 (22.8) *μ*m for Lava, 58.5 (17.6) *μ*m for Cercon, and 72.3 (30.8) *μ*m for Cerec. There were no significant differences in the marginal fit among the groups except that the Cercon crowns had significantly smaller marginal gaps than the Lava crowns (*P* < 0.001).  *Conclusions*. Within the limitation of this study, all the metal-free restorations made by the digital CAD/CAM systems had clinically acceptable marginal accuracy.

## 1. Introduction

With increasing demand for aesthetics, many studies on zirconia, which is the most representative element for metal-free restoration in the field of restorative dentistry, have been recently performed due to its acceptable aesthetics and high strength that is comparable with the strength of a metal ceramic crown [1–8]. Yttria-stabilized tetragonal zirconia polycrystal is provided as a block form to secure the maximum strength [6, 7]. A new precise mechanical subtracting process has been introduced instead of the previous adding method including waxing, investing, and casting to fabricate a prosthodontic shape from the block. A computer-aided design/computer-aided manufacturing (CAD/CAM) system has been further developed in dentistry over the last 20 years to handle very precise data acquisition, complex restoration design, complete task processing, and high-end cutting system [9].

One of the most important elements in evaluating a fixed prosthodontic device is marginal accuracy. Every prosthodontic restoration process, from abutment preparation to cementation, has effects on the marginal fit of the restoration [10]. Unlike the traditional analogue methods, the CAD/CAM system needs the precision of the system itself, including the accurate digital conversion of acquired information and calibration of the digitalized data according to materials used in CAM. Therefore, it is important in clinical CAD/CAM application to prosthodontic restoration to understand both the differences between the CAD/CAM systems and the accuracy of the resulting crowns.

This study aimed to investigate the marginal fit of zirconia crowns made by widely used CAD/CAM systems: Lava (3M ESPE, Seefeld, Germany), Cercon (DeguDent, Hanau, Germany), and Cerec (Sirona Dental Systems GmbH, Bensheim, Germany). This study also compared the marginal fit of the zirconia crowns with that of a metal ceramic gold (MCG) crown, which is one of the restoration forms clinically used for the longest period.

## 2. Materials and Methods

**Figure 1:**
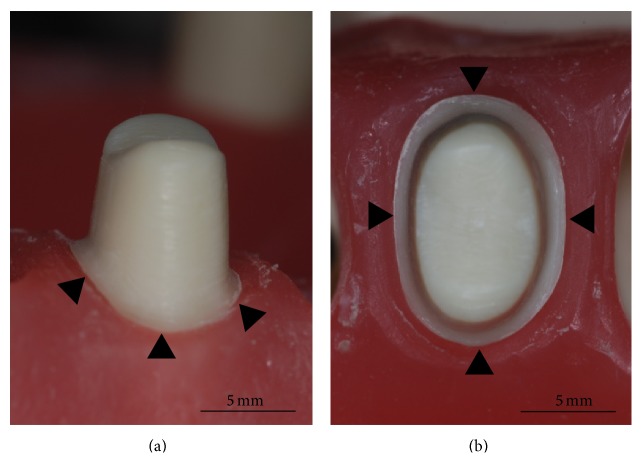
The prepared premolar resin tooth from the buccal (a) and occlusal viewpoints (b). Note the circumferential cervical margin of 1 mm width (black arrowheads).

The maxillary left first premolar (#24) of the dentiform (Columbia Dentoform Corp., New York) was prepared to form an abutment tooth. Two millimeters of the occlusal surface and 1.0–1.4 mm of the lateral side were reduced. The completed convergence angles of the abutment were about 8–10° both mesiodistally and buccolingually. The margin was assigned with 1 mm of a heavy chamfer margin in the overall range of the cervical aspect (Figure 1). After the abutment preparation, the resin tooth was invested onto the plaster, and the impression was acquired by using the additional silicone impression products of putty and light body (Exafine, GC Co., Tokyo, Japan). Forty original resin models (Exakto-Form, Bredent, Senden, Germany) were manufactured from the silicone impression. These resin models were subsequently used for the measurements of the marginal openings after the final restorations were cemented to these models. The models were divided into 4 groups by assigning 10 models to each group. Lava, Cercon, and Cerec systems were used to fabricate final restorations. Ten single MCG premolar crowns served as control, which were made by the conventional casting method. The other all-ceramic crowns were fabricated according to the manufacturers' recommendations of the systems evaluated in this study. The gap for cement was all assigned as 60 *μ*m.

**Figure 2:**
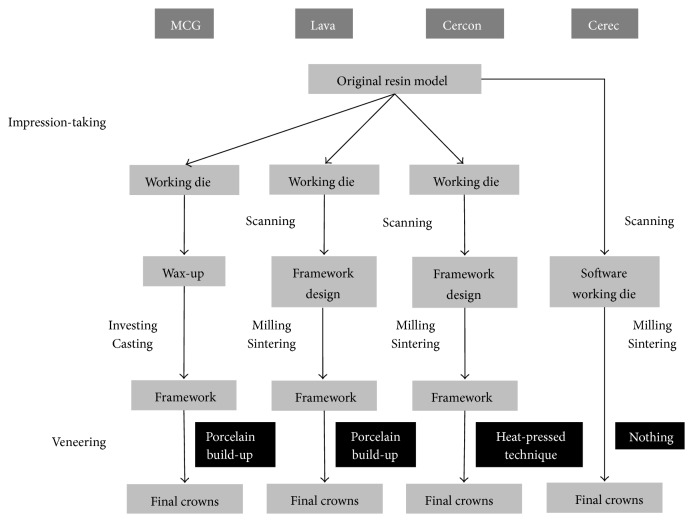
Summarized fabrication procedures for each system. Note that there was neither impression-taking nor veneering procedure in the Cerec system, which manufactured the purely digitalized crowns. Also, notice that each all-ceramic system used a different veneering technique. MCG; metal ceramic gold crowns.

The working die productions for the MCG, Lava, and Cercon crowns were performed using high strength dental stone (GC Fujirock EP, GC Europe N.V., Leuven, Belgium) after taking impressions of the original resin models with the additional silicone impression materials of putty and light body (Exafine). The virtual working dies for the Cerec crowns were produced by direct scanning method. For the production of MCG crowns, a wax pattern was produced by using a conventional method with high strength dental stone model. The die spacer (Pico-Fit Die Spacer Varnish (silver), Renfert USA, Il, USA) was coated 3 times on high strength dental stone. Considering the fact that 1 time die spacer coating creates a layer thickness of 14–20 *μ*m from the manufacturer's technical data, the practice allowed for a cement space of approximately 42–60 *μ*m. The gold (Bio Herador SG, Heraeus, Germany) coping was produced by following the investing and casting procedures and then veneered with porcelain. For the Lava crowns, the high strength dental stone dies were scanned with a scanner (Lava Scan Scanner) and zirconia copings were designed under a CAD system (Lava CAD), which gave the cement space of 60 *μ*m. The copings were produced by milling zirconia blocks (Lava zirconia blocks) with a CAM system (Lava Form Milling Unit). The copings were manufactured by setting the thickness of the coping at 0.5 mm. The final crowns were completed by veneering porcelain (Lave Ceram) on the copings after sintering. In the manufacturing of Cercon crowns, the working dies were also scanned using a scanner (Cercon EYE) and zirconia frameworks were designed using a CAD software (Cercon ART). Zirconia blocks (Cercon zirconia blocks) were then milled using a CAM system (Cercon BRAIN), to make the frameworks that were 0.5 mm thick. The milled zirconia frameworks were sintered and were veneered with a heat-pressed material (IPS e.max Ceram, Ivoclar Vivadent AG, Benderer Str. 2, Liechtenstein) and technique, to manufacture the final crowns. For the fabrication of Cerec crowns, the original resin models were directly scanned (CEREC Bluecam) to make the software working dies and the final crowns were designed using a CAD software (CEREC 3D). Zirconia blocks (IPS e.max ZirCAD, Ivoclar Vivadent AG, Schaan, Liechtenstein) were milled by a CAM system (CEREC in Lab MC XL milling machine) and sintered to make the final restorations with no veneering procedure. The procedures, instruments, and materials to make the specimens are summarized in Figure 2.

The MCG, Lava, Cercon, and Cerec crowns were, respectively, cemented to their own resin models by using a resin cement (RelyX Unicem Clicker, 3M ESPE, Germany). During cement setting time, 50 N loading was applied with finger pressure by the person who had trained to calibrate the 50 N load with a laboratory scale. Excessive cement material was cleaned with cotton pellets. The marginal fit of each sample was measured by using a stereoscopic microscope (Nikon DS-Fi 1, Nikon, Japan) at 75x magnification. The marginal gap was defined in this study as a distance on the microscope from a point of the tooth margin to the intersecting point between the restoration margin and the line perpendicular to the tangent line to the tooth margin at the tooth margin point. For each crown, the gap was measured at one point of the labial, lingual, mesial, and distal surface. The marginal gap of a crown was calculated as the mean of the measured four gaps.

The mean and standard deviation (SD) were calculated for the measured marginal gaps of each group. One-way ANOVA and a post hoc test, Duncan's test, were used to find any statistically significant difference among the groups at the level of significance of 0.05.

## 3. Results

The mean marginal gaps (SD) of MCG, Lava, Cercon, and Cerec crowns were determined to be 70.5 (34.4) *μ*m, 87.2 (22.8) *μ*m, 58.5 (17.6) *μ*m, and 72.3 (30.8) *μ*m, respectively. The descriptive statistics including the mean, SD, minimum, and maximum measured values of each group are presented in Table 1. One-way ANOVA and Duncan's post hoc test showed that there were no significant differences in the marginal fit among the groups except that the Cercon crowns had significantly smaller marginal gaps than the Lava crowns (*P* < 0.001).

## 4. Discussion

There are many and various criteria about the clinically acceptable marginal fit of prosthodontic restoration [11–15]. ADA specification number 8 defined that the range should be 25–40 *μ*m, and Ostlund stated that the value should not exceed 50 *μ*m [11]. Unfortunately, those values appear to be very difficult to obtain clinically. Christensen reported that a maximum marginal distance of 119 *μ*m was allowed by dentists for the proximal surface of gold inlays through observations using eyes, probes, and radiographic images and stated an approximate 39 *μ*m maximum marginal distance for the occlusion surface [12]. McLean and von Fraunhofer stated that a marginal gap of about 100 *μ*m does not cause any clinical problems in a study observing 1,000 dental restorations performed over more than 5 years, concluding that the clinically allowable maximum marginal discrepancy was 120 *μ*m [13]. Another previous study evaluated that a marginal gap up to 100 *μ*m was clinically acceptable, while still another extended the clinically acceptable marginal gap to 200 *μ*m [14, 15]. There is still controversy over the clinically acceptable marginal fit standard. However, most authors are considered to agree upon the fact that the marginal discrepancy should be less than 200 *μ*m [16–23].

The measurement values that were acquired in the present study were in the clinically acceptable range for all the test groups. Most of the currently used CAD/CAM systems were found to show appropriate clinical marginal fit by exhibiting a mean marginal discrepancy value of less than 200 *μ*m. Bindl and Mörmann found no significant difference in the marginal fit of crowns, when comparing the marginal fit of CAD/CAM all-ceramic crowns of Cerec inLab, DCS, Decim, and Procera, the slip cast type crown of In-Ceram zirconia, and heat-pressing type crown of Empress 2, showing a marginal opening range of about 20–70 *μ*m [24]. The marginal fit of the 4-unit fixed dental prostheses made by four CAD/CAM systems (Cercon, Cerec inLab, Digident, Everest) was evaluated to be 57.9–206.3 *μ*m [25]. Another previous study investigating the marginal accuracy of 3-unit fixed dental prostheses showed the mean marginal gaps of 77–92 *μ*m for the Cerec inLab, Digident, and Lava systems [26]. The previous results were similar to those of this study although there were some numerical differences according to the experimental conditions including the restored teeth (anterior, posterior), the restoration types (single, multiple), and the fabrication procedures.

The Cercon premolar crowns exhibited significantly superior marginal fit to the Lava crowns in this study. However, these statistics were unable to be interpreted as superiority of one system in precision to the other because there were no significant differences either between the Cercon and the control (MCG) groups or between Lava and control. Differences in the veneer techniques, not those in the CAD/CAM systems, could explain some causes of the results shown in this study. Some previous studies showed the differences in accuracy between the restorations with and without the porcelain build-up procedures and the significant effects of the veneering methods on restoration precision [27, 28]. This investigation, however, did not consider a CAD/CAM system and a veneer technique as two independent variables, which was one of the limitations. Further studies are required to evaluate and to compare the effects of those two factors, the systems and veneering methods, on the marginal accuracy of prosthodontic restorations. In addition, this study indicated that the accuracy of a dental restoration fabricated by digital technology may be clinically acceptable, when compared with that by conventional analogue method. However, various approaches were found according to the CAD/CAM systems: pure digital techniques and digital-analogue combinations, as shown in Figure 2. Further studies are needed to compare each step in digital procedures with that in analogue.

## 5. Conclusions

Computer-aided digital technologies may manufacture metal-free restorations that are clinically acceptable in precision. Considering the results in this study, the marginal gaps of the digitalized metal-free crowns were similar to those of the conventional metal ceramic gold crowns. All the accuracy investigated in this study may be within the generally agreed clinically acceptable marginal fit standard.

## Figures and Tables

**Table 1:** Mean, SD, and minimum and maximum values of the marginal fit for each of the groups.

Group	Mean (SD)	Minimum–maximum
MCG	70.5 (34.4)^AB^*∗*^^	31.9–207.7
Lava	87.2 (22.8)^B^	45.1–140.9
Cercon	58.5 (17.6)^A^	34.8–97.3
Cerec	72.3 (30.8)^AB^	21.8–164.1

^*∗*^The groups with the same superscript letters (A and B) were not significantly different (unit; *μ*m).
